# Possible Involvement of the Nutrient and Energy Sensors mTORC1 and AMPK in Cell Fate Diversification in a Non-Metazoan Organism

**DOI:** 10.3389/fcell.2021.758317

**Published:** 2021-11-08

**Authors:** Julian D. Gross, Catherine J. Pears

**Affiliations:** Department of Biochemistry, University of Oxford, Oxford, United Kingdom

**Keywords:** *Dictyostelium discoideum*, cell fate decision, AMP-dependent protein kinase (AMPK), mechanistic target of rapamycin complex 1 (mTORC1), ammonia, acidic vesicles, DIF-1

## Abstract

mTORC1 and AMPK are mutually antagonistic sensors of nutrient and energy status that have been implicated in many human diseases including cancer, Alzheimer’s disease, obesity and type 2 diabetes. Starved cells of the social amoeba *Dictyostelium discoideum* aggregate and eventually form fruiting bodies consisting of stalk cells and spores. We focus on how this bifurcation of cell fate is achieved. During growth mTORC1 is highly active and AMPK relatively inactive. Upon starvation, AMPK is activated and mTORC1 inhibited; cell division is arrested and autophagy induced. After aggregation, a minority of the cells (prestalk cells) continue to express much the same set of developmental genes as during aggregation, but the majority (prespore cells) switch to the prespore program. We describe evidence suggesting that overexpressing AMPK increases the proportion of prestalk cells, as does inhibiting mTORC1. Furthermore, stimulating the acidification of intracellular acidic compartments likewise increases the proportion of prestalk cells, while inhibiting acidification favors the spore pathway. We conclude that the choice between the prestalk and the prespore pathways of cell differentiation may depend on the relative strength of the activities of AMPK and mTORC1, and that these may be controlled by the acidity of intracellular acidic compartments/lysosomes (pHv), cells with low pHv compartments having high AMPK activity/low mTORC1 activity, and those with high pHv compartments having high mTORC1/low AMPK activity. Increased insight into the regulation and downstream consequences of this switch should increase our understanding of its potential role in human diseases, and indicate possible therapeutic interventions.

## Introduction

Individual starved amoebae of *D. discoideum* aggregate to form loose mounds, which are transformed into “tight aggregates”, in which the amoebae are closely associated via lateral and polar contacts and become surrounded by a complex extracellular matrix of protein and cellulose to create, in effect, a multicellular organism (Kessin, 2001; Pears and Gross, 2021). Once within the mounds, the cells become divided into two cell types, prestalk and prespore cells, defined by myriad differences in gene expression. The two types of cell are initially distributed in a salt and pepper manner within mounds (Thompson et al., 2004) but soon separate, with the prestalk cells moving first to the periphery of the mound and then to its apex; the aggregate then elongates to form a “first finger” or “standing slug” divided into a leading zone made up of prestalk (pst) cells, and a posterior zone of prespore (psp) cells. This standing structure can fall over onto the substratum and migrate for a period as a migratory slug or proceed immediately to form a fruiting body consisting of mature spores held up by a cellular stalk made up of stalk cells.

Early attempts to answer the question of how the divergence of cell type is achieved focused on the stalk cell-inducing factor DIF-1 (Town et al., 1976; Kay and Jermyn, 1983). DIF-1 is a low molecular weight chlorinated alkyl phenone that was purified from the conditioned medium of developing amoebae and induces isolated amoebae to differentiate into vacuolated stalk cells following pre-treatment with a high concentration of extracellular cyclic AMP (cAMP). A number of different models have been proposed in which DIF-1 plays a central role in controlling cell type divergence (Gross et al., 1981; Meinhardt, 1983; Gruenheit et al., 2018). Factors such as the glycogen content of amoebae grown in high glucose and the cell cycle phase at the onset of starvation are known to influence cell fate preference (McDonald and Durston, 1984; Weijer et al., 1984; Gomer and Firtel, 1987; Araki et al., 1994; Thompson and Kay, 2000a). These have been proposed to lead to differential sensitivity to DIF-1 induction of prestalk gene expression (Thompson and Kay, 2000a; Gruenheit et al., 2018). However, although mutants unable to synthesise or respond to DIF-1 display a number of developmental defects, in particular lacking one subtype of prestalk cell, they are able to form normal developmental structures such as first fingers, slugs and fruiting bodies with a roughly normal pattern of prestalk and prespore cells (Thompson and Kay, 2000b; Saito et al., 2008; Murata et al., 2016). Therefore, differential ability of cells to respond to DIF-1 cannot explain cell type divergence.

### Roles of AMPK and mTORC1 in Cell Fate Choice

The proposal we wish to make is based on the behavior of the nutrient and energy sensors mTORC1 (mechanistic target of rapamycin complex 1) and AMPK (AMP-activated protein kinase), and on the fact that *Dictyostelium* development takes place during starvation. The universal serine/threonine protein kinases mTORC1 and AMPK (Hardie, 2007; Saxton and Sabatini, 2017) have emerged as major nutrient sensors that control the responses to changes in nutrient and energy levels by activating complex downstream networks of signaling proteins such as other protein kinases and transcription factors. When eukaryotic cells are incubated in the presence of adequate concentrations of amino acids and glucose, mTORC1 is activated and AMPK activity is inhibited; cells multiply and autophagy is reduced*.* In response to starvation, mTORC1 activity declines and AMPK activity increases, resulting in arrest of cell division in G1 (Rattan et al., 2005) and the induction of autophagy which has the effect of conserving energy and obtaining a minimal level of nutrients from catabolism (Hardie, 2007; Jung et al., 2010; Saxton and Sabatini, 2017).

This response is conserved when amoebae of *D. discoideum* are starved: mTORC1 activity is rapidly down-regulated and AMPK activity increases (Rosel et al., 2012; Jaiswal and Kimmel, 2019); cell division and DNA replication soon cease, autophagosomes appear in increased numbers (Zimmerman and Weijer, 1993; King et al., 2011) and autophagy genes are upregulated (Jaiswal et al., 2019). At the same time, development is initiated and transcription switches from the growth transcriptome to the early aggregative transcriptome (Jaiswal and Kimmel, 2019). However, while cell division ceases at the onset of development, it recommences post aggregation in the posterior region of slugs containing the prespore cells [(Zimmerman and Weijer, 1993; Muramoto and Chubb, 2008) and references therein]. At the same time autophagic activity declines, but does not altogether cease, in the prespore population.

It occurred to us that this reversal of activities in the prespore cells could be the consequence of a reversal of the relative activities of AMPK and mTORC1 in these cells; in other words, that prespore gene expression might be initiated in the future prespore cells when AMPK activity was down-regulated and mTORC1 activity restored. It has already been proposed that the mutual inhibition between mTORC1 and AMPK constitutes a regulatory switch responsible for initiating development (Jaiswal and Kimmel, 2019), and our suggestion is just an extension of this idea to account for the bifurcation of cell fates. The most straightforward test of our proposal would be to follow the phosphorylation activities of AMPK and mTORC1 during early development to determine if AMPK activity declines and mTORC1 activity increases prior to the appearance of prespore cell-specific markers.

**FIGURE 1:**
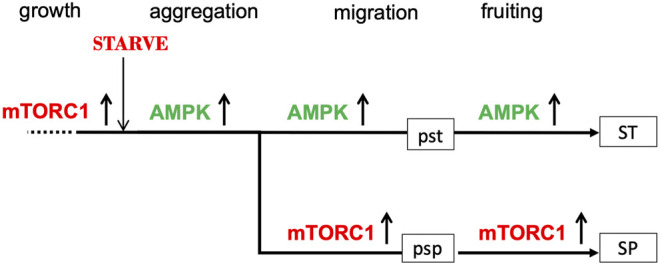
Suggested roles of AMPK and mTORC1 activities in the prestalk (pst) and prespore (psp) pathways. During growth, mTORC1 is active (upward arrow) and AMPK activity is low (not shown). Upon nutrient withdrawal, mTORC1 activity is rapidly switched off and AMPK activity upregulated (upward arrow); as a result, aggregative stage gene expression is turned on, growth is arrested and autophagy activated. At the mound stage, mTORC1 is re-activated in some 80% of the amoebae (and AMPK downregulated) and expression of the prespore transcriptome is initiated; AMPK continues to be highly active in the remaining 20% of amoebae, which eventually form vacuolated stalk cells. The prestalk pathway is represented by a continuous straight line with the prespore pathway diverging from it to emphasise the asymmetry of the proposed bifurcation process. Only the member of the AMPK/mTORC1 pair that is highly active at any one time is shown.

This hypothesis for the relationship between the pst and psp pathways envisages the stalk cell pathway as a continuous pathway starting from starvation and the onset of development and proceeding all the way through aggregation to the formation of vacuolated stalk cells (Figure 1). The spore pathway, on the other hand, is seen as *deviating from* the prestalk pathway. This asymmetry is consistent with the observation that many “prestalk-specific” genes are first expressed during the aggregation stage (Jermyn et al., 1987; Tsujioka et al., 2001) whereas prespore-specific genes tend to be first expressed after aggregation, at around 8–10 h into development (Iranfar et al., 2001). We refer to the point at which this deviation occurs as the bifurcation point. The spore pathway can include novel products not present during growth, such as the spore coat proteins, since cells entering the spore pathway contain transcriptional machinery made during the aggregation stage.

### Evidence for Stalk Pathway Control by AMPK

#### *In vivo* Stalk Cell Formation

If the choice of the prestalk pathway is controlled by the activation of AMPK we might expect that overexpressing AMPK would increase the ratio of prestalk: prespore cells, and an effect of this kind has been observed by several workers. AMPK, as its full name of 5′AMP-activated protein kinase indicates, is activated by an increase in the AMP:ATP ratio. Mutants lacking AMP deaminase cannot deaminate AMP (adenosine 5′-monophosphate) to IMP (inosine 5-monophosphate) and as a result accumulate abnormally high levels of AMP (Chae et al., 2002). These strains form fruiting bodies with short, thick stalks and fewer than 5% of the usual number of spores; levels of prestalk-specific mRNAs are more than two-fold higher than those in wild-type strains and prespore-specific mRNAs are reduced. Slugs formed by the mutant strain have twice the normal number of prestalk cells, and enlarged prestalk zones. Similarly, expression of a truncated (and hence deregulated) form of the *Dictyostelium* AMPK catalytic subunit to increase AMPK activity led to the same kind of “stalky” phenotype as over-expression of AMPK (Bokko et al., 2007). Similar developmental effects have been observed in strains with defective mitochondrial function, which is thought to lead to reduced ATP synthesis and an elevated AMP/ATP ratio, and so to hyper-activation of AMPK. However, we note that, in contradiction to the above findings, fruiting bodies from cells lacking the catalytic subunit of AMPK have been reported to have increased prestalk zones (Maurya et al., 2017). However, late developmental phenotypes were not complemented by re-expression of the relevant subunit. Also AMPK transcripts were found to be restricted to the pst cells of slugs (Maurya et al., 2017), supporting the idea that AMPK activity is downregulated in the prespore “compartment”.

#### *In vitro* Stalk Cell Formation in Response to DIF-1

**FIGURE 2:**
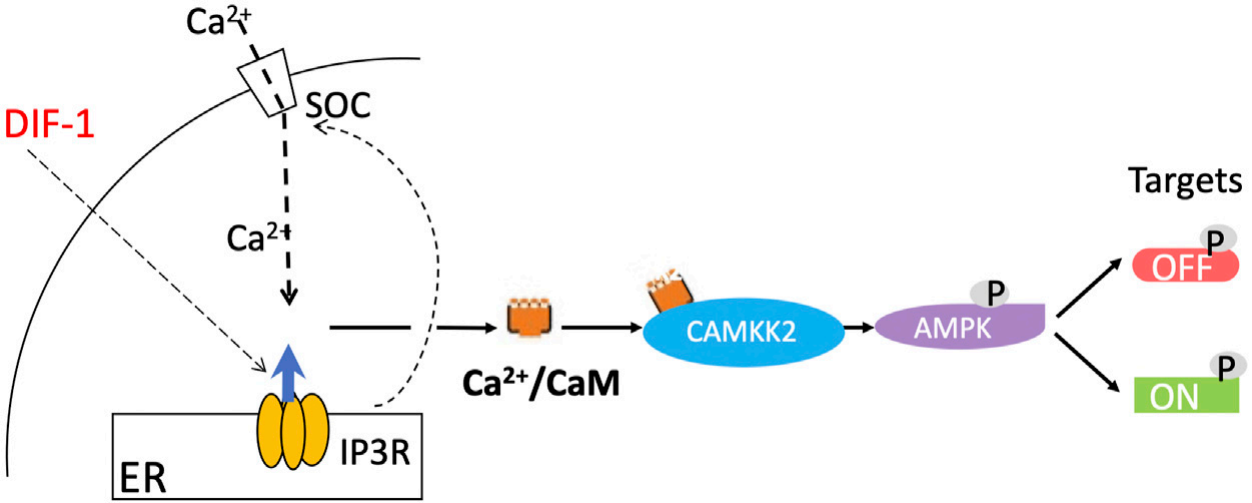
Proposed pathway of stalk cell induction by DIF-1. DIF-1 triggers an increase in cytosolic Ca^2+^, a process dependent on IplA, a protein related to the mammalian IP_3_ receptor, which triggers Ca^2+^ release from the endoplasmic reticulum (Traynor and Kay, 2017) and may also induce Ca^2+^ entry into the cell via a store-operated calcium channel. The concentration of Ca^2+^/calmodulin in turn increases, binding to and activating the calmodulin-dependent protein kinase CaMKK2. This then phosphorylates and activates AMPK, which phosphorylates various target proteins, activating some (including calcineurin and inhibiting others (Sugden et al., 2015) and initiating the prestalk/stalk (ST) pathway.

As mentioned, DIF-1 does not seem to play a decisive role in the initial cell fate choice in normal development and yet it dramatically stimulates stalk cell formation when cells are starved in monolayers in the presence of cAMP (Town et al., 1976). If AMPK controls the stalk pathway *in vivo* as we propose, we would expect it also to play a role in DIF-induced stalk cell formation. In agreement with this suggestion, exposure of *Dictyostelium* amoebae to DIF-1 leads to extensive changes in protein phosphorylation at sites many of which fit the AMPK consensus motif (Sugden et al., 2015), and DIF-1 activates AMPK in mouse 3T3-L1 fibroblasts (Kubohara et al., 2021). Also there is substantial evidence that DIF-1 acts by increasing cytosolic Ca^2+^, which is known in other systems to activate AMPK via the Ca^2+^/calmodulin-dependent protein kinase, CaMKK2 (Hurley et al., 2005) (Figure 2). Addition of DIF-1 has been shown to induce a rapid increase of intracellular Ca^2+^ (Azhar et al., 1997; Traynor and Kay, 2017), as well as a gradual and sustained rise in cytosolic Ca^2+^ (Schaap et al., 1996), and the stalk-cell inducing activity of DIF-1 is mimicked by the Ca^2+^-ATPase inhibitors BHQ and thapsigargin, which raise cytosolic Ca^2+^ by inhibiting sequestration of Ca^2+^ into intracellular stores (Schaap et al., 1996; Kubohara et al., 2007; Kubohara et al., 2021). Moreover DIF-1 fails to induce a rise in cytosolic Ca^2+^ in mutants lacking IplA, the putative endoplasmic reticulum IP3 receptor (Traynor and Kay, 2017), and also fails to induce stalk cells in these mutants (Lam et al., 2008).

### Evidence for Control of the Spore Pathway by mTORC1

Prespore-specific gene expression requires exposure to extracellular cAMP, which acts on cell surface receptors (cARs) [see (Yamada and Schaap, 2019)]. A provisional prespore pathway (Grimson et al., 2000) derived from the work of several groups and based on mutants with reduced proportions of prespore cells is:

cAMP receptor → Zak1 → GskA--→ Aar (a β-catenin homolog)→ psp gene expression.

For reasons that are not understood, mutations affecting this pathway have very variable effects on the extent of prespore and spore cell formation [see for example (Schilde et al., 2004)]. On the other hand, strains with disruption of the genes encoding any of these proteins that have been tested have the same effect in the monolayer differentiation system: they render stalk cell induction by DIF-1 insensitive to inhibition by the presence of cAMP (Harwood et al., 1995), because of partial or complete elimination of the psp pathway as a competitor to the stalk pathway (Early and Williams, 1988; Berks and Kay, 1990). A corollary of this is that stalk cell induction in these mutants is hypersensitive to DIF-1 (needs lower concentrations of DIF-1) when the assay is performed with cAMP present along with the DIF-1.

Until recently there was no evidence connecting the spore pathway with mTORC1. Any effect of mTORC1 could involve a parallel pathway or mTORC1 could influence the activity of the above pathway. There is clearly a complex interaction between mTORC1 and Gsk3 activity in humans which is not fully understood (Sun, 2021) and has been implicated in tumour resistance to mTORC1 inhibition. Recently a *Dictyostelium* mutant has been described in which the gene encoding 1-phosphatidylinositol-3-phosphate 5-kinase (PIKfyve) has been disrupted (Yamada et al., 2021), and properties of the mutant strain point to involvement of mTORC1 in the psp/spore pathway. PIKfyve converts PtdIns3P to PtdIns3,5P_2_ (PIP_2_), and there is strong evidence that PIP_2_ is located on the vacuole membrane of yeast and on acidic vesicle/endosomal membranes in mammalian cells and act as an anchor to associate mTORC1 to these membranes (Bridges et al., 2012), a precondition for its activation. The mutant has enlarged lower stalks; spore coat protein synthesis is initiated in Golgi-derived prespore vesicles as in the wild type, but mature spore formation is highly defective. Most importantly, *in vitro* observations show that the mutant is clearly DIF-1 hypersensitive, as defined above. Since this seems to be a characteristic of all mutants blocked in the spore pathway, it is consistent with the inference that PIKfyve, and, by extension, mTORC1, is a key player in that pathway. This conclusion could be tested by measuring mTORC1 activity in the mutant; also by seeing whether mTORC1 inhibitors render stalk cell induction in cell monolayers hypersensitive to DIF-1 in the presence of cAMP and block *in vitro* psp/spore formation in response to cAMP (in the absence of DIF-1).

In humans, the central roles of PIKfyve in endosomal trafficking and autophagy make it an attractive candidate for treatment of a number of diseases, using PIKfyve inhibitors such as apilimod. These diseases include cancer and neurodegenerative conditions as well as viral infections including those due to coronaviruses (Ikonomov et al., 2019; Huang et al., 2021). Understanding its interplay with the mTORC1 and AMPK pathways in *Dictyostelium* will have a bearing on the development of such inhibitor treatments.

### pHv and Symmetry Breaking

If we accept that the prespore pathway is initiated when AMPK is downregulated, and mTORC1 upregulated, in some 80% of the aggregated cells, what might be responsible for this switch? During growth and early in development, the acidic intracellular compartments of amoebae [although these are of various kinds, we will refer to them throughout as acidic vesicles (AVs)] are highly acidic and are stained by the weak base, neutral red, whereas at the first finger and slug stage of development, the AVs of prespore cells have lost their acidity and are unstained (Bonner, 1952). We suggest that this change in acidic vesicle pH (pHv) is responsible for downregulating AMPK and upregulating mTORC1 in these cells, although we cannot exclude the possibility that it is simply a consequence of their differences in gene expression. Support for the former possibility is based primarily on the finding that exposure of developing amoebae *in vitro* to weak bases switches them from the stalk to the spore pathway, whereas weak acids have the opposite effect (Gross et al., 1983; Kubohara and Okamoto, 1994) while at the same time altering the pH of their AVs (Yamamoto and Takeuchi, 1983; Marchetti et al., 2009). Although the differential neutral red staining pattern is sometimes not evident until slugs have migrated for some time, it is revealed within minutes by covering the slugs with mineral oil, which provides an impermeable barrier to the diffusion of ammonia (Feit et al., 1990). This effect can be explained if there is already a substantial difference in pHv between the cell types at the beginning of the experiment, but that for loss of neutral red staining of the prespore cells to occur, ammonia must accumulate in the AV’s of these cells to a level at which their pHv is above the pKa (pH6.7) of neutral red.

In contrast to the marked difference between prestalk and prespore cells in terms of pHv, their cytosolic pH values are either not different (Kay et al., 1986; Town et al., 1987) or differ by at most 0.2 pH units (Inouye, 1988), so the effects of pH on cell type choice are unlikely to depend on a difference in cytosolic pH. Exposure to 50 mM ammonia lowered the acidity of AVs by at least two orders of magnitude but had no detectable effect on cytosolic pH (Davies et al., 1993), thus identifying AVs as the likely target of weak base action. These effects are seen at the level of prestalk versus prespore gene expression, as well as in terms of the final mature cell types (Bradbury and Gross, 1989), and are most easily accounted for if vesicle pH controls the pathway of gene expression.

Further evidence that pHv influences cell fate choice comes from the remarkable 1-dimensional differentiation system devised by Bonner (Bonner et al., 1995). In this system an extracellular pH gradient is generated by spontaneous ammonia production (Sawai et al., 2002), and cells expressing a prespore gene appear at the alkaline end of the pH gradient while those expressing prestalk genes are found at the more acidic end (Sawai et al., 2002) where AV’s have been shown to be acidic (Azhar et al., 1996).

**FIGURE 3:**
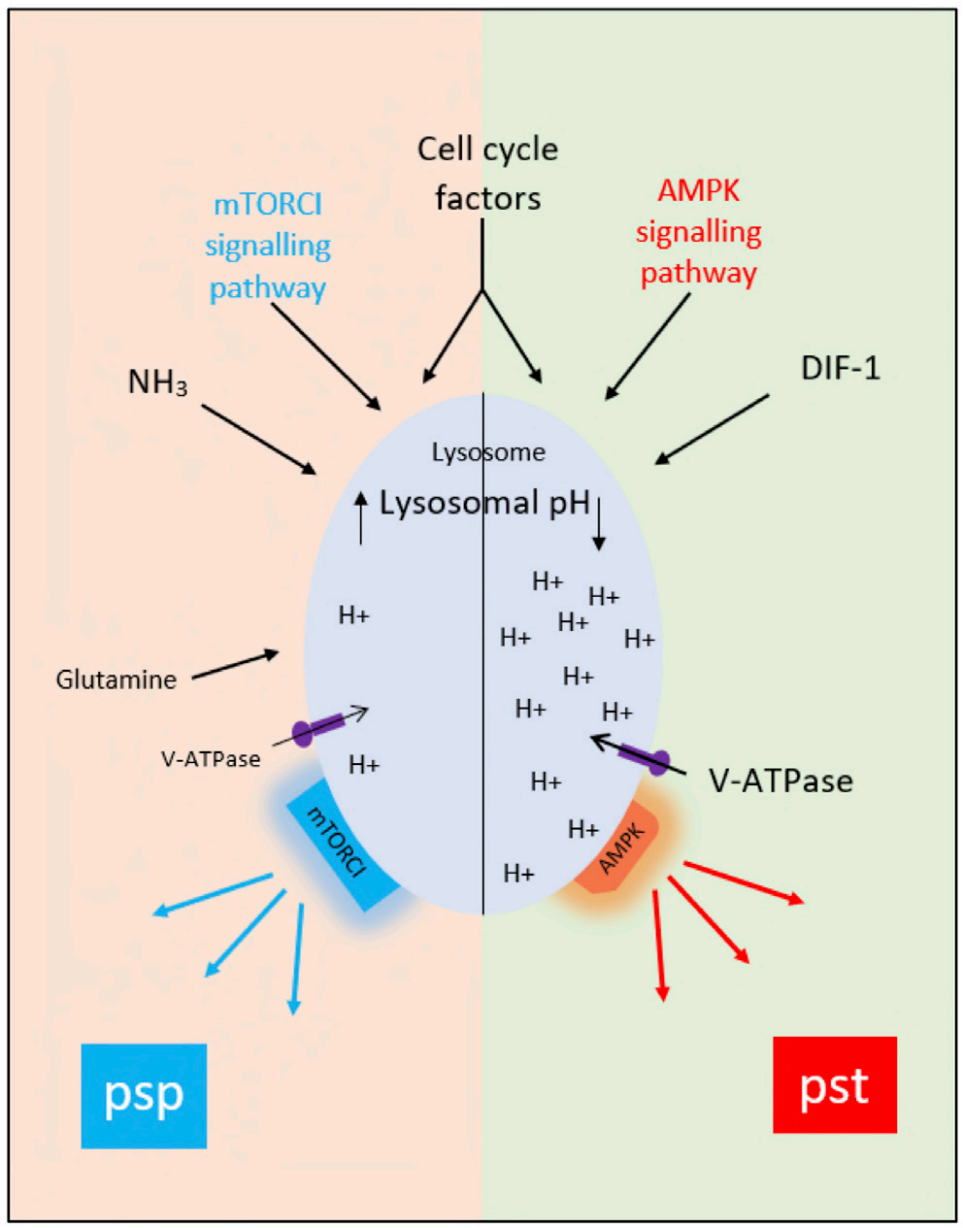
The proposed role of acidic vesicle pH (pHv) in control of the prestalk and prespore pathways at the mound stage of development. Multiple factors including AMPK and mTORC1, cell cycle stage at the time of starvation, glycogen content, V-ATPase activities, cell adhesion and cell movement probably contribute to the division of a population of aggregated amoebae into those with acidified acidic vesicles (right-hand side of diagram). and those with neutralised acidic vesicles (left-hand side of diagram). We suggest that AMPK remains activated in the former group of cells, leading to continued prestalk gene expression, whereas it is downregulated in the latter group of cells and mTORC1 activated, initiating prespore gene expression.

The idea that AV acidification status influences cell fate choice is not as strange as it might once have seemed since, in addition to their well-known activities in degradation and recycling, lysosomes are now seen to be organizing hubs regulating such processes as signaling, nutrient sensing, metabolic adaptation, organellar interactions and aging (Savini et al., 2019). Moreover, both AMPK and mTORC1 are thought to be present on lysosomes, and to be physically associated with the lysosomal V-ATPase responsible for acidifying the lysosomal lumen (Zhang et al., 2014) (Figure 3), potentially via PIP_2_ (Ho et al., 2012; Jin et al., 2014). In many cases activation of mTORC1 appears to require lysosomal acidification and/or V-ATPase activity (Laplante and Sabatini, 2012). In the case of *Dictyostelium*, the proposed association between loss of acidity and initiation of the prespore pathway at the bifurcation point would require that it is AMPK activity that is dependent on vesicle acidification. If that is the case, we would expect that reduced vesicle acidification at the point when amoebae are starved to initiate development would interfere with the activation of AMPK and the corresponding downregulation of mTORC1 activity. In agreement with this expectation, the loss of mTORC1 activity upon starvation is delayed in a *Dictyostelium* mutant defective in acidifying AVs (Chang et al., 2020). Interestingly, loss of presenilin genes in *Dictyostelium* has been shown to block development by reducing vesicular acidification (Sharma et al., 2019). Mutations in presenilins in humans associated with Alzheimer’s disease also result in elevated lysosomal pH and defects in autophagy (Lee et al., 2010). Other neurodegenerative disorders such as Parkinson’s disease also show alterations in this pathway (Nixon, 2013). Thus, understanding the mechanisms and consequences of alterations in pHv in *Dictyostelium* will have an impact on our understanding of Alzheimer’s disease and other neurogenerative diseases and on the eventual development of treatments.

An alternative interpretation of the mode of action of ammonia has been proposed to explain its well-known inhibitory effect on cAMP levels (Singleton et al., 1998), in which ammonia transporters act as ammonia sensors activating or inhibiting the hybrid histidine kinase DhkC in response to the local ammonia level; the level of DhkC activation would then be relayed to the RegA cAMP phosphodiesterase which would in turn be activated or inhibited. The authors discount a role for vacuolar pH in this process. While a role for DhkC in the cAMP response to ammonia levels has been established, it seems unlikely that the ammonia transporters are acting directly as sensors, since many other weak bases affect cAMP levels in the same way as ammonia and are not substrates of the ammonia transporters. Moreover, mutants in the V-ATPase itself cause the same developmental defects as exposure to weak bases (including prolonged slugging) (Davies et al., 1996), which would not be the case if these defects were due to ammonia transport activity. Instead, we suggest that defects in vesical acidification have two, probably independent, consequences: on the one hand they result in activation of the DhkC phosphorelay and breakdown of cAMP, on the other they inhibit AMPK activation (and/or promote mTORC1 activation).

The question remains of how the cells of aggregates become split into those with acidified vesicles and those with neutralized vesicles. One mechanism for creating such a division would be a process of local self-activation and lateral inhibition (Meinhardt, 1983), with ammonia as inhibitor, taking place within small random groups of cells in cell aggregates. However, the sorting behavior of mutants defective in vesicle acidification casts doubt on this idea. We would expect that on a lateral diffusion model, cells defective in acidification would sort preferentially to the prespore region in mixtures with wild type cells, since that is the region occupied by cells with neutralized AVs; instead, they show a remarkable preferential localization in the anterior, acidified, compartment (Kirsten et al., 2008; Sharma et al., 2019). This indicates that pHv influences cell fate choice in *some* way, perhaps associated with the cell cycle, since cell fate is also strongly influenced by the stage of the division cycle at which cells are starved (McDonald and Durston, 1984; Araki et al., 1994; Huang et al., 1997; Jang and Gomer, 2011; Gruenheit et al., 2018) and other factors (Figure 3). Thus, the mechanism controlling differences in pHv appears to be complex and is elusive.

## Discussion

Gene expression in response to cAMP signaling in *Dictyostelium* is thought to involve two processes: firstly activation of poorly understood intracellular pathways by extracellular cAMP signals acting on cell surface G protein-coupled cAMP receptors, and secondly activation of cAMP-dependent protein kinase by cAMP synthesized within the cell by the actions of three distinct adenylate cyclases. According to our proposal, cell-type specialization, with its accompanying differential gene expression*,* is controlled by the opposing actions of mTORC1 and AMPK acting on this background of cAMP signaling. mTORC1 and AMPK, are often viewed as predominantly controlling responses to variations in food and energy supplies by phosphorylating cytoplasmic factors such as p70S6K and 4E-BP1. If those were their only targets it would be hard to see how they could control differential gene transcription on the two differentiation pathways. However, both agents are now known to also have profound effects on cell type-specific gene transcription in mammalian cells (Laplante and Sabatini, 2012; Sukumaran et al., 2020). The genome of *D. discoideum* contains at least 245 genes predicted to encode transcription factors*,* more than half of which are restricted to either prestalk or prespore cells (Forbes et al., 2019). Of these 245 transcription factors, just 34 have been examined in gene disruption or knockdown experiments, and nine of them caused defects in one specific cell type. There would therefore be ample opportunity for phosphorylation by mTORC1 and AMPK to control the differential production and/or activities of particular transcription factors and thus differential gene transcription in the two pathways. Interestingly, in the similar system involving the choice between adipocyte and osteocyte differentiation in human mesenchymal stem cells, which is controlled by mTORC1 and AMPK (Chen et al., 2017), recent work has revealed a network of transcription factors and enhancers that control adipogenesis (Rauch et al., 2019).

As well as this choice between the osteoblast and adipocyte fates of mesenchymal stem cells (Chen et al., 2017), many other cell fate choices in metazoans are also controlled by the mutually antagonistic effects of mTORC1 and AMPK. This applies for example to alternative T cell differentiation pathways in response to antigen (Chi, 2012), and the control of ageing in both yeast and higher organisms (Hindupur et al., 2015). Similarly, the inflammatory cells activated by pathogens and tissue damage are mTOR-dependent while anti-inflammatory cells are AMPK-dependent (O'Neill and Hardie, 2013). However, although the bifurcation of cell fates in *Dictyostelium* as we envisage it would reflect the same mutual antagonism between AMPK and mTORC1, it is unlike those situations in that it takes place under starvation where mTORC1 is not normally activated. In fact, according to our suggestion, prespore/spore pathway cells would resemble more closely tumour cells since in both mTORC1 activation is uncoupled from normal regulatory nutrient signals.

There is another possible parallel between tumour cells and prespore pathway cells. Paradoxically, many tumours, though fundamentally mTORC1-dependent and not dependent on extensive autophagic activity nevertheless rely on a low level of autophagy for their growth and spread, and hence on residual AMPK activity (given that autophagy depends on AMPK activity) (Kimmelman and White, 2017). Autophagy is thought to supply nutrients to these tumours and confer resistance to stressors such as starvation, anti-cancer drugs etc, and clinical trials are currently underway to test inhibition of autophagy as an anticancer strategy (Perez-Hernandez et al., 2019). Interestingly, a strikingly similar observation to the situation in those tumours is found in the case of the *Dictyostelium* prespore pathway (Yamada and Schaap, 2019); mutations such as *atg7* that reduce the efficiency of autophagy virtually eliminate prespore gene expression while not affecting prestalk gene expression. This can be understood in terms of our basic proposal if we suppose that autophagy, while an essential component of the prestalk pathway, is also needed at a low level in the prespore pathway; it should therefore be fully activated by AMPK in the pst pathway but substantially downregulated by mTORC1 in the prespore pathway. Consequently, if the efficiency of autophagy is reduced by a mutation such as *atg7* this will virtually eliminate autophagy in the prespore pathway and block prespore gene expression, while leaving the prestalk pathway almost intact.

Understanding the mechanisms and consequences of mTORC1 regulation in *Dictyostelium* has implications for treatment of a range of human diseases. For example, ketogenic diets, which involve intake of high levels of fat and low levels of carbohydrate, were developed to mimic starvation (Augustin et al., 2018). Such diets are used to treat seizures although recently it has been suggested that their mode of action is not due to the diet itself but to a direct effect of medium chain fatty acids such as decanoic acid, a major dietary breakdown product of diglycerides (Chang et al., 2013; Warren et al., 2018). Studies in *Dictyostelium* have demonstrated that decanoic acid inhibits mTORC1 activity, independently of glucose and insulin signaling (Warren et al., 2020). This work has identified a novel pathway to regulate mTORC1 activity. Decanoic acid inhibits mTORC1 activity in mammalian cells including astrocytes from tuberous sclerosis complex (TSC) patients. This syndrome is caused by mutations in TSC1 or TSC2, resulting in epilepsy, cognitive disfunction and behavioral abnormalities. Individuals with this neurodevelopmental disorder have hyperactive mTORC1. This work in *Dictyostelium* therefore points to decanoic acid as a promising therapy. Conversely cannabidiol (CBD), a major component of cannabis oil, enhances mTORC1 activity in *Dictyostelium* in a mechanism dependent on inositol polyphosphate multikinase (Damstra-Oddy et al., 2021). This pathway has been verified in mammalian cells and shown to increase mTORC1 activity in primary peripheral blood mononuclear cells from patients suffering from multiple sclerosis. This work initiated in *Dictyostelium* identifies CBD as a potential therapy for multiple sclerosis through increased mTORC1 activity. DIF-1 inhibits the growth of a range of different cancer cells (Takahashi-Yanaga et al., 2014; Kubohara et al., 2015; Arioka et al., 2017). The mechanisms of action is as yet ill-defined although there is a recent report that it supresses the growth of triple negative breast cancer cells by a mechanism involving AMPK-dependent inhibition of mTORC1 activity (Seto-Tetsuo et al., 2021) in a pathway reminiscent of the cell fate decision pathway we are proposing in *Dictyostelium*. It will be of interest to define the role of the pH of acidic vesicles in the effect of DIF-1 on growth of these cancer cells as alteration of this may offer an alternative therapy route.

In conclusion, *Dictyostelium* may provide a tractable non-metazoan model for examining in detail how the two ancient sensor molecules, mTORC1 and AMPK, control the differential gene expression underlying a cell fate choice.

## Data Availability

The original contributions presented in the study are included in the article/Supplementary Material, further inquiries can be directed to the corresponding author.
